# Association between transient appetite loss and vitamin B1 deficiency in elderly patients with suspected deficiency

**DOI:** 10.1002/jgf2.404

**Published:** 2020-11-17

**Authors:** Ryuichi Ohta, Yoshinori Ryu, Shuzo Hattori

**Affiliations:** ^1^ Community Care Unnan City Hospital Unnan Japan; ^2^ Internal Medicine Unnan City Hospital Unnan Japan

**Keywords:** appetite loss, elderly patients, Japan, rural, vitamin B1 deficiency

## Abstract

**Background:**

There is scarce evidence associating vitamin B1 levels and appetite loss duration in elderly patients with suspected B1 deficiency. We aimed to investigate this association in elderly hospitalized patients with suspected vitamin B1 deficiency in rural Japan.

**Methods:**

This cross‐sectional study evaluated 309 elderly patients (aged ≥ 65 years) admitted to one rural Hospital between April 2017 and March 2019. We collected data on vitamin B1 level, age, sex, body mass index, albumin levels, area of residence, long‐term care, dependent conditions, activities of daily living, Charlson comorbidity index, and appetite loss from the patients' electronic medical records. Vitamin B1 deficiency was defined as serum vitamin B1 levels <20 μg/dL. Data were analyzed using the Mann‐Whitney *U*, Student's *t*, and chi‐square tests, followed by multivariable logistic regression, to examine the association between vitamin B1 deficiency and appetite loss.

**Results:**

Eighty‐eight (28.5%) patients had vitamin B1 deficiency. In multivariable logistic regression, appetite loss (for both < 1 and > 1 week) before admission to the hospital showed a significant association with vitamin B1 deficiency (adjusted odds ratio [AOR] =10.80, 95% confidence interval [CI]: 5.16‐22.00, *P* < .001; and AOR = 5.77, 95% CI: 2.88‐11.50, *P* < .001, respectively).

**Conclusions:**

Appetite loss is associated with vitamin B1 deficiency in elderly Japanese patients living in rural areas. Therefore, physicians should be aware of the possibility of vitamin B1 deficiency in elderly patients with appetite loss and focus on early intervention.

## Graphical abstract

This study clarifies the positive association between the short duration of appetite loss and vitamin B1 deficiency among older patients in rural Japan

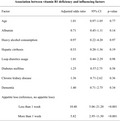

## 1 INTRODUCTION

Vitamin B1 malabsorption can be a serious problem in elderly patients, causing various diseases, and ultimately resulting in death.^1^, ^2^ Absorption of vitamin B1 can be hindered because of multiple medical problems, such as atrophy of the gastrointestinal mucus membrane and low intake of vitamin B1‐rich foods.^3^ Vitamin B1 acts as a coenzyme for various metabolic reactions in the body. Therefore, inadequate blood vitamin B1 levels can cause several metabolic abnormalities leading to various symptoms and diseases, such as wet and dry beriberi and deterioration of cognitive functions.^4^ Medical problems related to vitamin B1 deficiency can be corrected by identifying the underlying etiology and promptly providing suitable treatments.^1^

It may be challenging to modify food intake, which is influenced by patients' culture, lifestyle, background, and eating habits.^5^, ^6^ Similarly, the intake of vitamin B1 is also influenced primarily by the residential location, eating habits, and age of an individual.^7^ In rural areas, patients tend to consume plant‐based foods, such as rice and vegetables.^8^ Although some of these foods may contain vitamin B1, the method of cooking may decrease its availability for intestinal absorption, thereby resulting in its deficiency.^9^, ^10^ Aging can also reduce the absorption of vitamin B1 because the atrophied gastrointestinal tract absorbs vitamins with lower efficiency.^11^ Additionally, elderly patients tend to have various underlying conditions owing to multimorbidity, which affects their gastrointestinal environment.^12^, ^13^ These conditions, in turn, can lead to appetite loss, causing a decrease in the blood levels of vitamin B1.

Early detection of vitamin B1 deficiency can help to initiate appropriate treatment, especially in hospitals. Indeed, prompt supplementation of vitamin B1 could prevent the deterioration of physiological and cognitive functions in hospitalized patients.^2^, ^14^ Moreover, patients with acute symptoms may experience appetite loss, which can increase the risk of vitamin B1 deficiency.^15^ However, there is little evidence relating vitamin B1 levels and appetite loss duration in elderly patients with suspected vitamin B1 deficiency.^16^ Despite rice being abundant in rural mountainous areas, older adults in these areas consume processed rice and vegetables and limited varieties of red meat, which contribute to the increased risk of vitamin B1 deficiency.^17^ Additionally, the initial suspected diagnosis of vitamin B1 deficiency could depend on the physicians' ability and experience.^16^ Clarification of the association between vitamin B1 deficiency and duration of appetite loss can help in the appropriate diagnosis of elderly patients with suspected vitamin B1 deficiency.

We aimed to determine the association between vitamin B1 deficiency and the appetite loss duration among elderly patients with suspected vitamin B1 deficiency in a rural community hospital in Japan.

## 2 MATERIALS AND METHODS

### 2.1 Setting

A rural city is one of the most rural cities in Japan, located in the southeast region of Shimane prefecture. In 2017, the total population of the rural city was 38 882 (18 720 men and 20 162 women). The proportion of city residents aged >65 years was 37.82%. In the rural city, there is only one public hospital. During the study period, the rural city hospital had 281 beds comprising 160 acute care, 43 comprehensive care, 30 rehabilitation, and 48 chronic care beds.^18^

### 2.2 Participants

Study participants were selected among all the patients admitted to our institution. The selection criteria included age >65 years and the presence of initial findings suggestive of vitamin B1 deficiency, assessed by general or internal medicine physicians on the day of admission based on physicians' chart. The patients' data were collected between April 2017 and March 2019 from the electronic medical records of our institution.

### 2.3 Data collection

The presence of vitamin B1 deficiency was considered a dependent variable for this study. Vitamin B1 deficiency was defined as serum vitamin B1 levels <20 μg/dL. The risk factors for vitamin B1 deficiency were based on previous research and were evaluated as independent variables; the data of these variables were also collected from electronic medical records.^14^, ^15^ These variables included appetite loss duration before admission (<1 week to >1 week),^15^ age, sex, albumin levels, body mass index (BMI), admission from a long‐term care facility, dependent conditions based on the Japanese long‐term care insurance system, activities of daily living (ADL),^19^ history of aspiration, tube feeding or parenteral nutrition, heavy alcohol consumption (>80 g/d), Charlson comorbidity index (CCI),^20^ heart failure, dementia, diabetes mellitus, hyperthyroidism, hepatic cirrhosis, malignancy, and the use of loop diuretics. Appetite data were collected based on patients' and families' expressions and the nurses' descriptions concerning the patients' appetite and the duration of appetite loss since admission. Patients were subsequently classified into the "appetite loss" (<1 week to >1 week) and "no appetite loss" groups, as it is estimated that the duration of vitamin B1 stores in humans is approximately 7 days.^15^ Cases without available descriptions of appetite were excluded from this study.

### 2.4 Statistical analysis

For continuous variables, the normality of the data was tested before applying the statistical tests. The parametric and nonparametric data were analyzed using Student's *t* test and the Mann‐Whitney *U* test, respectively. The chi‐square test was used for testing the categorical data. The following continuous variables were dichotomized for the logistic regression model: level of vitamin B1 (>20 μg/dL or not), ADL (Katz scale score = 6 or not), and CCI (>6 or not). A multivariable logistic regression analysis was conducted to examine the association between vitamin B1 deficiency and other factors, including appetite loss. All variables that were factors known to be associated with vitamin B1 deficiency, as well as those variables that were found to be significant in the univariable regression model for vitamin B1 deficiency, were used to construct the multivariable logistic regression models. Regarding the sample size calculation, 229 participants would be needed with 80% statistical power and 5% type 1 error to detect a difference in the percentage of patients with vitamin B1 deficiency of 20% between the appetite loss and no appetite loss groups. Cases with missing data were eliminated from the analysis. All data analyses were performed using Easy R, version 1.23 (R Foundation for Statistical Computing, Vienna, Austria). Statistical significance was defined at *P* < .05.

### 2.5 Ethical considerations

The hospital was assured of no loss of anonymity and confidentiality of the patients' information. The information related to this study was posted on the hospital website without disclosing any details concerning the patients. To address any questions regarding this study, the contact information of the hospital representative was also listed on the Web site. The purpose of this study was explained to all patients, and informed consent was obtained. The Clinical Ethics Committee of our institution approved this study.

## 3 RESULTS

### 3.1 Demographics of the participants

**Figure 1:**
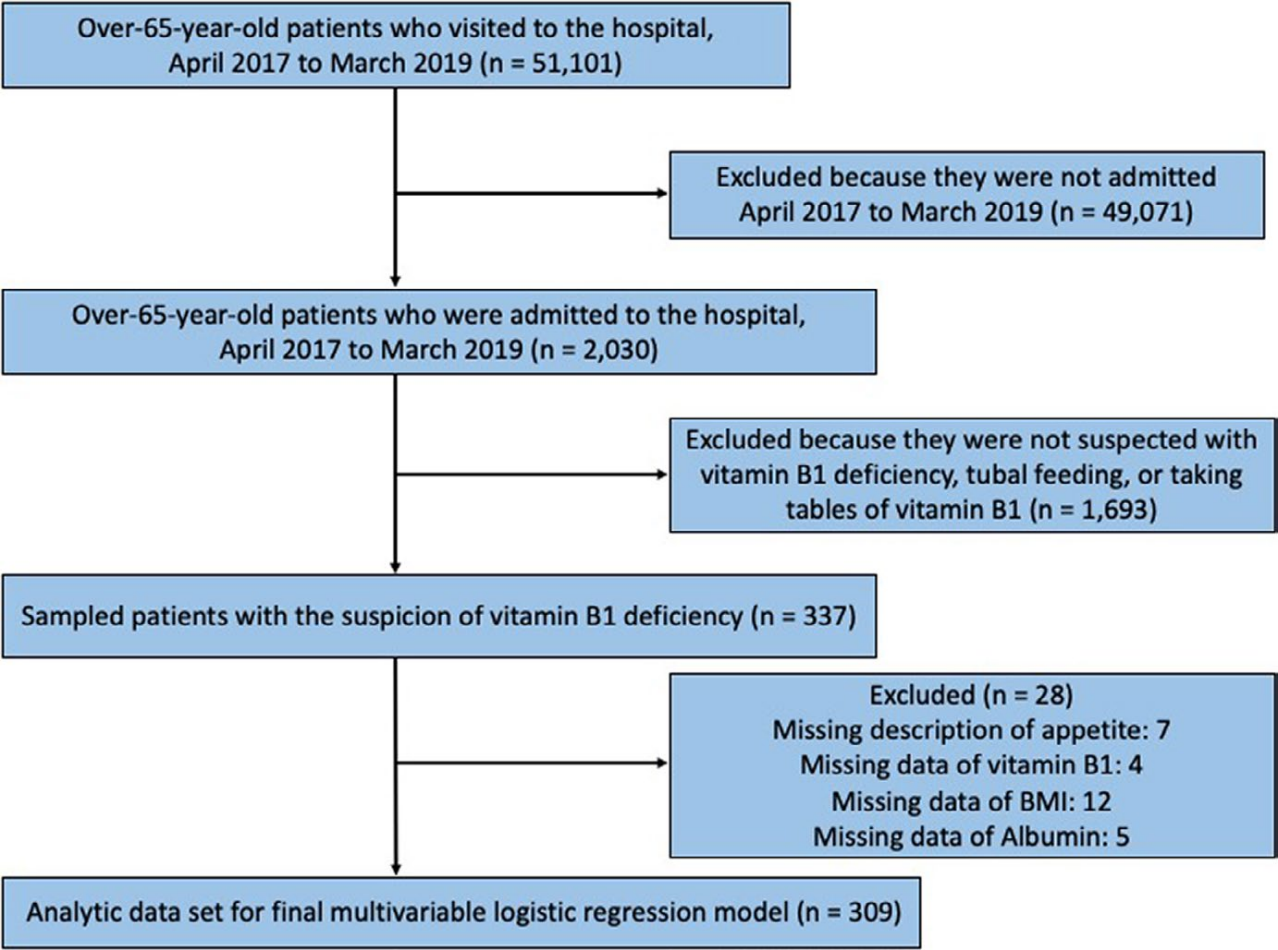
Flowchart of the study population selection process

Figure 1 shows the flowchart of the study population selection process. Between April 2017 and March 2019, 51 101 patients aged >65 years visited the outpatient departments of general or internal medicine. Among them, 2030 were admitted to the hospital. We included a total of 337 participants with suspected vitamin B1 deficiency, based on their medical record data. Twenty‐eight patients were excluded because of the lack of description regarding their appetite conditions, data regarding vitamin B1 concentration before admission, and data regarding other independent variables (Figure 1).

Eighty‐eight (28.5%) out of the 309 participants had vitamin B1 deficiency. The average patient age in the vitamin B1‐deficient and nondeficient groups was 87.07 (standard deviation [SD] = 7.62) and 85.02 (SD = 7.90) years, respectively. Age, albumin levels, and appetite loss were significantly different between the two groups (Table 1). There was no patient with hyperthyroidism. The most common diagnosis was heart failure, followed by bacterial pneumonia, urinary tract infection, brain stroke, and cancer (Table 2).

**Table 1:** Demographics of the study participants

Characteristics	Vitamin B1 deficiency		*P*‐value
	− (n = 221)	+ (n = 88)	
Age, mean (SD)	85.02 (7.90)	87.07 (7.62)	.039
Sex, male (%)	111 (50.2)	41 (46.6)	.459
Albumin, mean (SD)	3.41 (0.65)	3.19 (0.63)	.008
BMI, mean (SD)	19.35 (3.46)	19.05 (4.15)	.516
Dependent condition (%)	110 (49.8)	48 (54.5)	.077
Appetite loss (%)			
No	167 (75.6)	25 (28.4)	<.001
Less than 1 wk	22 (9.9)	32 (36.4)	
More than 1 wk	31 (14.0)	31 (35.2)	
Katz score (≥6) (%)	74 (33.5)	33 (37.5)	.51
CCI score (≥6) (%)	34 (38.6)	63 (28.5)	.103
Dementia (%)	44 (19.9)	27 (30.7)	.051
Diabetes (%)	32 (14.5)	15 (17.0)	.6
Heart failure (%)	65 (29.4)	28 (31.8)	.344
Chronic kidney disease (%)	55 (24.9)	27 (30.7)	.319
Hepatic cirrhosis (%)	28 (12.7)	8 (9.1)	.597
Malignancy (%)	33 (14.9)	13 (14.8)	1
Heavy alcohol consumption (%)	12 (5.4)	3 (3.4)	.568
History of aspiration (%)	30 (13.6)	15 (17.0)	.476
Transfer from a long‐term care facility (%)	36 (16.3)	16 (18.2)	.737
Use of loop diuretics (%)	30 (13.6)	13 (14.8)	.856

Abbreviations: BMI, body mass index; CCI, Charlson comorbidity index; N, number of patients; SD, standard deviation.

**Table 2:** Participants' diagnosis on admission

Number	Disease	Number of cases	Rate	Number	Disease	Number of cases	Rate
1	Heart failure	33	10.7%	20	Hyperglycemia	4	1.3%
2	Pneumonia	32	10.4%	21	Sepsis	4	1.3%
3	UTI	31	10.0%	22	Alcoholic disorder	3	1.0%
4	Brain stroke	22	7.1%	23	Cellulitis	3	1.0%
5	Cancer	21	6.8%	24	Guillain‐Barré syndrome	3	1.0%
6	Aspiration pneumonia	15	4.9%	25	Hyponatremia	3	1.0%
7	Femoral neck fracture	10	3.2%	26	Ileus	3	1.0%
8	Dehydration	7	2.3%	27	Vitamin B1 deficiency	3	1.0%
9	Epilepsy	7	2.3%	28	Cholangitis	2	0.6%
10	Compression fracture	6	1.9%	29	Hypoglycemia	2	0.6%
11	Pseudogout	6	1.9%	30	Hypothermia	2	0.6%
12	Brain hemorrhage	5	1.6%	31	IgG4 disease	2	0.6%
13	Peripheral vertigo	5	1.6%	32	Interstitial pneumonia	2	0.6%
14	COPD	4	1.3%	33	Ischemic colitis	2	0.6%
15	Hydrocephalus	4	1.3%	34	ITP	2	0.6%
16	Influenza	4	1.3%	35	MI	2	0.6%
17	LSS	4	1.3%	36	Nephrotic syndrome	2	0.6%
18	PMR	4	1.3%	37	Parkinson disease	2	0.6%
19	Gastric ulcer	4	1.3%	38	Others	39	14.9%

Abbreviations: COPD, chronic obstructive pulmonary disease; ITP, immune thrombocytopenic purpura; LSS, lumbar spinal stenosis; PMR, polymyalgia rheumatica; MI, myocardial infarction; UTI, urinary tract infection.

### 3.2 Association between vitamin B1 deficiency and influential factors

On multivariable logistic regression analysis, appetite loss (for both < 1 week and > 1 week) before admission to the hospital showed a significant association with vitamin B1 deficiency (adjusted odds ratio [AOR] = 10.40, 95% confidence interval [CI]: 5.06‐21.20, *P* < .001; and AOR = 5.82, 95% CI: 2.95‐11.50, *P* < .001, respectively). Age, albumin levels, heavy alcohol consumption, hepatic cirrhosis, loop diuretics usage, diabetes, chronic kidney diseases, and dementia showed no significant association with vitamin B1 deficiency (Table 3).

**Table 3:** Association between vitamin B1 deficiency and influencing factors

Factor	Adjusted odds ratio	95% CI	*P*‐value
Age	1.01	0.97‐1.05	.77
Albumin	0.71	0.45‐1.11	.14
Heavy alcohol consumption	0.97	0.22‐4.20	.97
Hepatic cirrhosis	0.53	0.20‐1.36	.19
Loop diuretics usage	1.01	0.44‐2.29	.98
Diabetes mellitus	1.25	0.57‐2.75	.58
Chronic kidney disease	1.36	0.71‐2.62	.36
Dementia	1.40	0.71‐2.75	.34
Appetite loss (reference, no appetite loss)			
Less than 1 wk	10.40	5.06‐21.20	<.001
More than 1 wk	5.82	2.95‐11.50	<.001

Abbreviation: CI: confidence interval.

## 4 DISCUSSION

This single‐center retrospective study investigated the association between vitamin B1 deficiency and appetite loss duration among elderly Japanese patients. The results showed that elderly patients admitted to rural community hospitals may be deficient of vitamin B1 at the time of admission. Moreover, the appetite loss duration before admission can be related to vitamin B1 deficiency in these patients. Therefore, when dealing with elderly patients with appetite loss, physicians should be aware of a possible vitamin B1 deficiency.

Vitamin B1 deficiency is prevalent among elderly patients in rural areas. In this study, one‐fourth of the patients had vitamin B1 deficiency. The high rate of vitamin B1 deficiency in our study could be due to the age of participants. Atrophy of the gastrointestinal mucus membrane owing to aging can affect the absorption of vitamin B1 in the gastrointestinal tract.^11^ Additionally, various chronic diseases and conditions, such as hepatic cirrhosis, chronic kidney disease, and diabetes that cause atherosclerosis and low secretion of gastric acid, can affect the absorption of vitamin B1.^7^, ^21^ The high incidence of atrophic gastritis among elderly Japanese people could explain the high prevalence of vitamin B1 deficiency in this population, following even a short duration of appetite loss.^22^ Studies evaluating the association between vitamin B1 deficiency and atrophic gastritis are, therefore, needed in the future.

Given the focus on nutrition today, vitamin B1 deficiency is thought to be nonprevalent. However, as aging can affect various metabolic pathways, there is a possibility of its recurrence. Our findings can serve as the basis for further research on the etiologies and prevalence of vitamin B1 deficiency among elderly patients.

We demonstrated that appetite loss for up to 1 week could cause vitamin B1 deficiency in hospitalized elderly patients with acute symptoms. Elderly individuals have various underlying conditions because of multimorbidity and polypharmacy,^13^ and they tend to visit hospitals more frequently than younger people.^23^ Moreover, vitamin B1 deficiency can induce various symptoms that mimic dementia, delirium, and other mental disorders.^3^ Therefore, elderly patients with unexplained symptoms and appetite loss should be evaluated for vitamin B1 deficiency. In this study, the AOR of appetite loss <1 week was higher than that of appetite loss >1 week. This result could be affected by the patients' and their families' ability to recall, as they might only state the duration they could recall. Moreover, patients with short appetite loss durations might have more severe symptoms and appetite loss, as they might have arrived at the hospital faster than patients with longer durations of appetite loss. Future studies should investigate the associations between multiple, unexplained symptoms, including appetite loss and vitamin B1 deficiency, and the precise severity of appetite loss, such as the amount of food intake.

The effectiveness of early interventions for vitamin B1 deficiency is well known. Various studies have demonstrated the efficacy of vitamin B1 supplementation in patients with specific diseases. Patients with heart failure need treatment with diuretics, which can cause vitamin B1 deficiency.^24^ Interestingly, vitamin B1 supplementation has been shown to improve cardiac function in these patients.^25^, ^26^ Moreover, vitamin B1 deficiency can induce delirium and Wernicke encephalopathy with vague symptoms in hospitalized patients.^27^ These diseases can be prevented or cured with appropriate vitamin B1 supplementation.^14^, ^28^ Therefore, elderly patients with heart failure and other chronic diseases should be monitored for their vitamin B1 levels. If vitamin B1 deficiency is suspected in elderly patients, general physicians should consider administering vitamin B1 injections and consult physicians specializing in gastroenterological medicine owing to the possibility of malabsorption. Mainly, when elderly patients are at a high risk of delirium or when they experience appetite loss, vitamin B1 supplements should be promptly administered.

This study had some limitations. The first limitation was the selection bias. The participants were chosen among patients admitted to our hospital who had findings suggestive of vitamin B1 deficiency and were tested for their vitamin B1 levels upon admission. Because of the lack of random sampling, there could have been a selection bias. In future investigations, the participant selection should be conducted prospectively and randomly. Second, nutritional issues can be related to food cultures across different regions. This study was conducted in rural Japan, where most elderly people eat white rice and a limited variety of red meats, which could have affected the results. As there is scarce research on vitamin B1 deficiency in different communities, future studies should investigate its prevalence among elderly people in different communities. Third, we used data regarding the history of patients and their families, as recorded by nurses, to assess the patients' appetite instead of seeking a direct description from the patients, which could also reveal inaccuracies in assessing the extent of appetite loss. Patients with acute diseases had various symptoms and often did not clearly state (the presence of) appetite loss. Future studies can reveal appetite loss duration and the relationship between vitamin B1 deficiency and the amount of appetite loss, in quantity, to provide a more precise definition of appetite loss.

In conclusion, this study shows that appetite loss is associated with vitamin B1 deficiency in elderly patients with acute symptoms. Therefore, medical professionals should be aware of the possibility of vitamin B1 deficiency in elderly patients suffering from appetite loss. Future studies could investigate the effectiveness of vitamin B1 supplementation in elderly patients with appetite loss immediately after hospitalization.

## CONFLICT OF INTEREST

The authors declare no conflict of interest for this article.

## References

[1] Fattal‐Valevski A . Thiamine (vitamin B1). J Evid Based Complementary Altern Med. 2011;16(1):12–20.

[2] Huang YC , Lee MS , Wahlqvist ML . Prediction of all‐cause mortality by B group vitamin status in the elderly. Clin Nutr. 2012;31(2):191–8. 22071291 10.1016/j.clnu.2011.10.010

[3] Huertas‐González N , Hernando‐Requejo V , Luciano‐García Z , Cervera‐Rodilla JL . Wernicke's encephalopathy, wet beriberi, and polyneuropathy in a patient with folate and thiamine deficiency related to gastric phytobezoar. Case Rep Neurol Med. 2015;2015:e624807. 10.1155/2015/624807 PMC4677180 26697247

[4] Rapala‐Kozik M . Vitamin B1 (Thiamine): a cofactor for enzymes involved in the main metabolic pathways and an environmental stress protectant. In: Rébeillé F , Douce R , editors. Advances in Botanical Research, vol. 58. New York, NY: Academic Press; 2011: 37–91.

[5] Morelli L . Yogurt, living cultures, and gut health. Am J Clin Nutr. 1248S;99:1248S–50S. 10.3945/ajcn.113.073072 24695895

[6] Tsuji T , Fukuwatari T , Sasaki S , Shibata K . Urinary excretion of vitamin B1, B2, B6, niacin, pantothenic acid, folate, and vitamin C correlates with dietary intakes of free‐living elderly, female Japanese. Nutr Res. 2010;30(3):171–8. 20417877 10.1016/j.nutres.2010.02.001

[7] Assantachai P , Lekhakula S . Epidemiological survey of vitamin deficiencies in older Thai adults: implications for national policy planning. Public Health Nutr. 2007;10(1):65–70. 17212845 10.1017/S136898000720494X

[8] Whitfield KC , Smith G , Chamnan C , Karakochuk CD , Sophonneary P , Kuong K , et al. High prevalence of thiamine (vitamin B1) deficiency in early childhood among a nationally representative sample of Cambodian women of childbearing age and their children. PLoS Negl Trop Dis. 2017;11(9):e0005814. 28873391 10.1371/journal.pntd.0005814 PMC5600402

[9] Blitshteyn S . Vitamin B1 deficiency in patients with postural tachycardia syndrome (POTS). Neurol Res. 2017;39(8):685–8. 28531358 10.1080/01616412.2017.1331895

[10] Muthayya S , Hall J , Bagriansky J , Sugimoto J , Gundry D , Matthias D , et al. Rice fortification: an emerging opportunity to contribute to the elimination of vitamin and mineral deficiency worldwide. Food Nutr Bull. 2012;33(4):296–307. 23424896 10.1177/156482651203300410

[11] Kiela PR , Ghishan FK . Physiology of intestinal absorption and secretion. Best Pract Res Clin Gastroenterol. 2016;30(2):145–59. 27086882 10.1016/j.bpg.2016.02.007 PMC4956471

[12] Heuberger RA , Caudell K . Polypharmacy and nutritional status in older adults. Drugs Aging. 2011;28(4):315–23. 21428466 10.2165/11587670-000000000-00000

[13] Mannucci PM , Nobili A , Investigators REPOSI . Multimorbidity and polypharmacy in the elderly: lessons from REPOSI. Intern Emerg Med. 2014;9(7):723–34. 25164413 10.1007/s11739-014-1124-1

[14] Pourhassan M , Angersbach B , Lueg G , Klimek CN , Wirth R . Blood thiamine level and cognitive function in older hospitalized patients. J Geriatr Psychiatry Neurol. 2018;32(2):90–6. 30572755 10.1177/0891988718819862

[15] Descombes E , Hanck AB , Fellay G . Water soluble vitamins in chronic hemodialysis patients and need for supplementation. Kidney Int. 1993;43(6):1319–28. 8315945 10.1038/ki.1993.185

[16] Pourhassan M , Biesalski HK , Angersbach B , Lueg G , Klimek C , Wirth R . Prevalence of thiamine deficiency in older hospitalized patients. Clin Interv Aging. 2018;2(13):2247–50. 10.2147/CIA.S183102 PMC6220429 30464433

[17] Shibata K , Fukuwatari T , Imai E , Hayakawa T , Watanabe F , Takimoto H , et al. Dietary reference intakes for Japanese 2010: water‐soluble vitamins. J Nutr Sci Vitaminol. 2012;59(Supplement):S67–82.

[18] Unnan City Hospital . [updated and cited, 2 Sep 2020]. Available from http://unnan-hp.jp/

[19] Restrepo A . The Katz activities of daily living scale. Am J Nurs. 1999;99(1):24BB.

[20] Charlson ME , Pompei P , Ales KL , MacKenzie CR . A new method of classifying prognostic comorbidity in longitudinal studies: development and validation. J Chronic Dis. 1987;40(5):373–83. 3558716 10.1016/0021-9681(87)90171-8

[21] de Torres Rossi RG , Dos Santos MT , de Souza FI , de Cássia de Aquino R , Sarni ROS . Nutrient intake of women 3 years after Roux‐en‐Y gastric bypass surgery. Obes Surg. 2012;22(10):1548–53. 22688468 10.1007/s11695-012-0688-y

[22] Asaka M , Sugiyama T , Nobuta A , Kato M , Takeda H , Graham DY . Atrophic gastritis and intestinal metaplasia in Japan: results of a large multicenter study. Helicobacter. 2001;6(4):294–9. 11843961 10.1046/j.1523-5378.2001.00042.x

[23] Moore L , Deehan A , Seed P , Jones R . Characteristics of frequent attenders in an emergency department: analysis of 1‐year attendance data. Emerg Med J. 2009;26(4):263–7. 19307386 10.1136/emj.2008.059428

[24] Ahmed M , Azizi‐Namini P , Yan AT , Keith M . Thiamin deficiency and heart failure: the current knowledge and gaps in literature. Heart Fail Rev. 2015;20(1):1–11. 24811895 10.1007/s10741-014-9432-0

[25] DiNicolantonio JJ , Niazi AK , Lavie CJ , O'Keefe JH , Ventura HO . Thiamine supplementation for the treatment of heart failure: a review of the literature. Congest Heart Fail. 2013;19(4):214‐22. 23910704 10.1111/chf.12037

[26] Schoenenberger AW , Schoenenberger‐Berzins R , der Maur CA , Suter PM , Vergopoulos A , Erne P . Thiamine supplementation in symptomatic chronic heart failure: a randomized, double‐blind, placebo‐controlled, cross‐over pilot study. Clin Res Cardiol. 2012;101(3):159–64. 22057652 10.1007/s00392-011-0376-2

[27] Ota Y , Capizzano AA , Moritani T , Naganawa S , Kurokawa R , Srinivasan A . Comprehensive review of Wernicke encephalopathy: pathophysiology, clinical symptoms and imaging findings. Jpn J Radiol. 2020;38(9):809–20. 32390125 10.1007/s11604-020-00989-3

[28] Osiezagha K , Ali S , Freeman C , Barker NC , Jabeen S , Maitra S , et al. Thiamine deficiency and delirium. Innov Clin Neurosci. 2013;10(4):26–32. PMC3659035 23696956

